# The Impact of Mask Mandates on Face Mask Use During the COVID-19 Pandemic: Longitudinal Survey Study

**DOI:** 10.2196/42616

**Published:** 2023-01-11

**Authors:** Mawuena Binka, Prince Asumadu Adu, Dahn Jeong, Nirma Khatri Vadlamudi, Héctor Alexander Velásquez García, Bushra Mahmood, Terri Buller-Taylor, Michael Otterstatter, Naveed Zafar Janjua

**Affiliations:** 1 British Columbia Centre for Disease Control Vancouver, BC Canada; 2 School of Population and Public Health University of British Columbia Vancouver, BC Canada; 3 Faculty of Pharmaceutical Sciences University of British Columbia Vancouver, BC Canada; 4 Faculty of Medicine University of British Columbia Vancouver, BC Canada; 5 Centre for Health Outcomes and Evaluation St Paul’s Hospital Vancouver, BC Canada

**Keywords:** face mask, face covering, COVID-19, SARS-CoV-2, outbreak, public health, health policy, trend analysis, logistic regression

## Abstract

**Background:**

Face mask use has been associated with declines in COVID-19 incidence rates worldwide. A handful of studies have examined the factors associated with face mask use in North America during the COVID-19 pandemic; however, much less is known about the patterns of face mask use and the impact of mask mandates during this time. This information could have important policy implications, now and in the event of future pandemics.

**Objective:**

To address existing knowledge gaps, we assessed face mask usage patterns among British Columbia COVID-19 Population Mixing Patterns (BC-Mix) survey respondents and evaluated the impact of the provincial mask mandate on these usage patterns.

**Methods:**

Between September 2020 and July 2022, adult British Columbia residents completed the web-based BC-Mix survey, answering questions on the circumstances surrounding face mask use or lack thereof, movement patterns, and COVID-19–related beliefs. Trends in face mask use over time were assessed, and associated factors were evaluated using multivariable logistic regression. A stratified analysis was done to examine effect modification by the provincial mask mandate.

**Results:**

Of the 44,301 respondents, 81.9% reported wearing face masks during the 23-month period. In-store and public transit mask mandates supported monthly face mask usage rates of approximately 80%, which was further bolstered up to 92% with the introduction of the provincial mask mandate. Face mask users mostly visited retail locations (51.8%) and travelled alone by car (49.6%), whereas nonusers mostly traveled by car with others (35.2%) to their destinations—most commonly parks (45.7%). Nonusers of face masks were much more likely to be male than female, especially in retail locations and restaurants, bars, and cafés. In a multivariable logistic regression model adjusted for possible confounders, factors associated with face mask use included age, ethnicity, health region, mode of travel, destination, and time period. The odds of face mask use were 3.68 times greater when the provincial mask mandate was in effect than when it was not (adjusted odds ratio [aOR] 3.68, 95% CI 3.33-4.05). The impact of the mask mandate was greatest in restaurants, bars, or cafés (mandate: aOR 7.35, 95% CI 4.23-12.78 vs no mandate: aOR 2.81, 95% CI 1.50-5.26) and in retail locations (mandate: aOR 19.94, 95% CI 14.86-26.77 vs no mandate: aOR 7.71, 95% CI 5.68-10.46).

**Conclusions:**

Study findings provide added insight into the dynamics of face mask use during the COVID-19 pandemic. Mask mandates supported increased and sustained high face mask usage rates during the first 2 years of the pandemic, having the greatest impact in indoor public locations with limited opportunity for physical distancing targeted by these mandates. These findings highlight the utility of mask mandates in supporting high face mask usage rates during the COVID-19 pandemic.

## Introduction

The rapid spread of SARS-CoV-2 worldwide led to the declaration of a global pandemic by the World Health Organization in March 2020 [1,2]. SARS-CoV-2 infection causes COVID-19, which, in extreme cases, results in severe lung damage, multiorgan failure, and death. Person-to-person spread of SARS-CoV-2 is mediated through aerosolized droplets that are generated during activities such as talking, singing, coughing, or sneezing [1,3,4]. When worn appropriately, face masks and other face coverings limit the spread of aerosolized droplets by trapping them within their fibers [5,6]. The utility of face masks and other face coverings in reducing person-to-person transmission of SARS-CoV-2 has been demonstrated in epidemiological and laboratory-based studies, as well as in real-world settings [5,7-12]. This efficacy, alongside the widespread availability and ease of use of face masks has prompted public health officials worldwide to advocate for, or mandate, face mask use in indoor public spaces and in settings with limited opportunity for physical distancing, as part of efforts to control the spread of the virus [13,14].

Public health mandates provide a blanket order for the application of interventions to reduce disease transmission rather than providing a choice for the adoption of those interventions. Hence, these mandates may be perceived as infringing on freedom of choice in those settings. Consequently, mask mandates and recommendations were met with resistance from certain groups [15,16]. The lack of consensus among global political and public health leaders on the need for face masks during the early stages of the COVID-19 pandemic, as well as misinformation and disinformation regarding the utility of masks and potential adverse effects of face mask use, may also explain this resistance [14,16-19]. Specific reasons for the lack of face mask use in a survey conducted among participants from several Western countries included discomfort, difficulty breathing, and skepticism about the ability of face masks to prevent infection [20]. Nevertheless, mask mandates and recommendations have contributed to decreased incidence of COVID-19 cases and related deaths worldwide [21-23]. The advent of COVID-19 vaccines and increasing vaccination coverage has prompted the relaxation of mask mandates and recommendations in various countries worldwide [13,24]. However, recent resurgences in COVID-19 cases in regions where mask mandates were rescinded [25-27] underscore the continued need for the use of face masks in certain regions as global vaccination efforts progress and more is learned about the efficacy of current vaccines in reducing the transmission of new and highly contagious variants. Understanding factors associated with face mask use and quantifying the impact of mask mandates is, therefore, important for health communication and decision-making by public health leadership, especially in the context of repeated outbreaks. Recent studies investigating the factors associated with nonuse of face masks in Canada provided much needed information on the motivation and belief systems underlying face mask use in the country [28,29]. However, limited information is available on face mask usage patterns, with and without provincial mask mandates, during the COVID-19 pandemic in Canada. This information could have important policy implications, now and for future respiratory virus-driven pandemic(s). In this study, we bridge this knowledge gap by assessing face mask usage patterns in the presence and absence of the provincial mask mandate and the factors associated with mask use among respondents of a population-based survey in British Columbia (BC), Canada.

## Methods

### Context

Initial public health measures to control the spread of COVID-19 were introduced in BC, Canada, on March 18, 2020 [30]; however, the provincial mask mandate requiring face masks in all indoor public spaces did not come into effect until November 19, 2020 [31,32]. Nevertheless, major retail locations in the province mandated the use of face masks between July and August 2020 [33], prior to the provincial mask mandate, as did BC public transit on August 24, 2020 [34]. Due in part to increasing COVID-19 vaccination rates, the provincial mask mandate was lifted on July 1, 2021, although the mandatory requirement for face mask use remained in effect at major retail locations [35]. The provincial mask mandate was reinstated for select indoor public places on August 25, 2021, remaining in effect until March 11, 2022 [36-38]. By April 8, 2022, all other public health requirements, including proof of vaccination for admission to certain locations, were no longer mandated [38].

### Study Population and Variable Definitions

The BC COVID-19 Population Mixing Patterns (BC-Mix) survey is an ongoing web-based survey developed to assess population mixing patterns during the COVID-19 pandemic among BC residents [39]. The survey, launched on September 4, 2020, is composed of 94 questions across six key domains: (1) demographic information; (2) COVID-19 testing and results, symptoms, and health behaviors; (3) activities and behavior in and outside of the home; (4) internet and social media use; (5) perceptions and attitudes around COVID-19; and (6) COVID-19 vaccine acceptance (added March 8, 2021). It is administered on the Qualtrics platform to English-speaking persons aged ≥18 years and residing in BC. Anonymous links to the survey were circulated via advertisements placed on Google and social media platforms, namely Instagram, Facebook, WhatsApp, YouTube, and Twitter. Detailed descriptions of survey design, domains, and recruitment methods have been published elsewhere [39]. Participants completed a baseline survey (for first-time respondents), and those who consented were invited to complete shorter follow-up surveys every 2 to 4 weeks.

This analysis was restricted to the baseline responses received between September 4, 2020, and July 31, 2022. Survey respondents who left home at least once the previous day (survey question: “How many times did you leave your home [or property, apartment] yesterday?”) were asked whether or not they used a face mask (“Did you use a face mask yesterday?”). Survey respondents who provided valid answers to the face mask question (“Yes,” “No,” or “Prefer not to answer”) were included in this analysis. As people who left their homes either did or did not wear a mask, those who answered “Prefer not to answer” either did not want to anonymously report not wearing masks or did not want to report wearing masks to researchers for some reason. Assuming the former formed the majority of this subgroup and wanting to capture as many types of nonusers of face masks as possible, responses to the face mask use question were recategorized as “Yes” and “No” (“No” + “Prefer not to answer”) for the purpose of this study. Other questions addressed ethnicity, education, employment status, location of face mask use, duration of face mask use, number of trips outside the home, distance travelled, destination, and mode of travel (Table S1 in the Multimedia Appendix 1). Time period was grouped by calendar month; thus, the periods during which the provincial mask mandate were in effect were defined as from November 2020 to June 2021 and from September 2021 to February 2022.

### Statistical Analyses

Descriptive analyses were done with and without sampling weights. Sampling weights were based on age, sex, geography (Health Authority region), and ethnicity; derived with a weighting adjustment technique [40] using available participant and BC 2016 Canadian Census data; and applied so that response frequencies were representative of the BC population. All comparisons between face mask users (face mask use=“Yes”) and nonusers of face masks (face mask use=“No”) were made with weighted data. Chi-square tests were used to ascertain between-group differences in variable distribution. Factors associated with face mask use were assessed with a multivariable logistic regression model, adjusting for time period, age group, sex, ethnicity, destination, number of trips taken, distance travelled, mode of travel, and Health Authority of residence—incorporating sampling weights. The association between the provincial mask mandate and face mask use was also assessed with multivariable logistic regression models, and a stratified analysis was done to examine effect modification by the mandate.

Data preparation, descriptive analyses, and data visualization were done with R statistical software (version 3.5.2; R Foundation for Statistical Computing) [41]. Weighted logistic regression modeling was done with SAS statistical software (version 9.4; SAS Institute) [42]. Statistical significance was assessed at the *P*<.05 level.

### Ethical Approval

This study complied with the ethical standards of the Helsinki Declaration. Participation was voluntary and electronic informed consent was sought from all participants on the survey start page. Analytical data sets were deidentified and included no personally identifiable information. Ethical approval for this study was provided by the University of British Columbia Behavioral Research Ethics Board (H20-01785).

## Results

### Respondent Characteristics

A total of 44,301 respondents were eligible for inclusion in this analysis (see Table 1). Survey respondents who answered the face mask question were mostly male (52.4%), not part of a visible minority group (63.3%), aged 25-34 years (18.81%) and 45-54 years (18.6%), employed full time (33%), and residing in the Fraser Health region (26.3%; Table 1).

**Table 1:** Proportion of British Columbia COVID-19 Population Mixing Patterns (BC-Mix) survey respondents by face mask use (yes, n=36,716; no, n=7585), stratified by demographic characteristics, from September 2020 to July 2022.

Characteristic		Unweighted values			Weighted values (distribution “across” groups)					*P* value^a^		Weighted values (distribution “within” each group)		
		Yes, n	No, n	Yes, n		No, n	Yes, %	No, %			Yes, %		No, %	
**Age group (years)**														
	18-24	1095	239	3244		822	8.4	9.6	.25		79.8		20.2	
	25-34	3459	696	7160		1673	18.6	19.6			81.1		18.9	
	35-44	5157	1066	6122		1385	15.9	16.2			81.6		18.4	
	45-54	6409	1159	7216		1553	18.7	18.2			82.3		17.7	
	55-64	9149	1969	6543		1370	17	16.1			82.7		17.3	
	65-74	8817	1886	6216		1324	16.1	15.5			82.4		17.6	
	≥75	2630	570	2008		411	5.2	4.8			83		17	
**Sex**														
	Female	29,833	5737	19,052		3362	49.5	39.4	<.001		85		15	
	Male	6883	1848	19,455		5176	50.5	60.6			79		21	
**Ethnicity**														
	Chinese	891	114	4298		541	11.2	6.3	<.001		88.8		11.2	
	South Asian	604	69	3186		395	8.3	4.6			89		11	
	Other visible minority	1267	155	2586		326	6.7	3.8			88.8		11.2	
	Not a visible minority	30,894	6439	23,922		5865	62.1	68.7			80.3		19.7	
	Other ethnicity	1916	430	3076		820	8	9.6			78.9		21.1	
	Prefer not to answer	1144	378	1441		591	3.7	6.9			70.9		29.1	
**Education**														
	Below high school	592	106	820		154	2.1	1.8	<.001		84.2		15.8	
	Below bachelor’s degree	13,834	2946	13,096		2997	34	35.1			81.4		18.6	
	University degree	13,597	2676	14,558		2839	37.8	33.2			83.7		16.3	
	Prefer not to answer or missing	8693	1857	10,034		2549	26.1	29.9			79.7		20.3	
**Employment status**														
	Employed full time	10,402	1758	12,975		2544	33.7	29.8	<.001		83.6		16.4	
	Employed part time	2807	427	2767		434	7.2	5.1			86.4		13.6	
	Self-employed	2268	562	2522		650	6.5	7.6			79.5		20.5	
	Unemployed	992	231	1459		362	3.8	4.2			80.1		19.9	
	Full-time parent or homemaker	648	199	535		176	1.4	2.1			75.3		24.7	
	Retired	9893	2339	6684		1456	17.4	17.1			82.1		17.9	
	Student or pupil	475	94	1029		220	2.7	2.6			82.4		17.6	
	Long-term sickness or disabled	670	126	654		137	1.7	1.6			82.7		17.3	
	Prefer not to answer or missing	8561	1849	9882		2559	25.7	30			79.4		20.6	
**Occupation**														
	Essential workers	7990	1536	9303		2161	24.2	25.3	<.001		81.2		18.8	
	Nonessential workers	10,246	1898	10,518		1949	27.3	22.8			84.4		15.6	
	Others	2458	500	2756		577	7.2	6.8			82.7		17.3	
	Do not work	7282	1749	5751		1206	14.9	14.1			82.7		17.3	
	Prefer not to answer or missing	8740	1902	10,180		2645	26.4	31			79.4		20.6	
**Health Authority**														
	Fraser Health	7339	1333	10,335		2060	26.8	24.1	<.001		83.4		16.6	
	Interior Health	4896	1354	4252		1382	11	16.2			75.5		24.5	
	Northern Health	1378	356	1441		479	3.7	5.6			75.1		24.9	
	Vancouver Coastal Health	7079	1103	7835		1218	20.3	14.3			86.5		13.5	
	Vancouver Island Health	7274	1574	4851		1096	12.6	12.8			81.6		18.4	
	Missing	8750	1865	9794		2303	25.4	27			81		19	

^a^Chi-square test.

### Face Mask Usage Patterns and the Impact of the Provincial Mask Mandate

**Figure 1:**
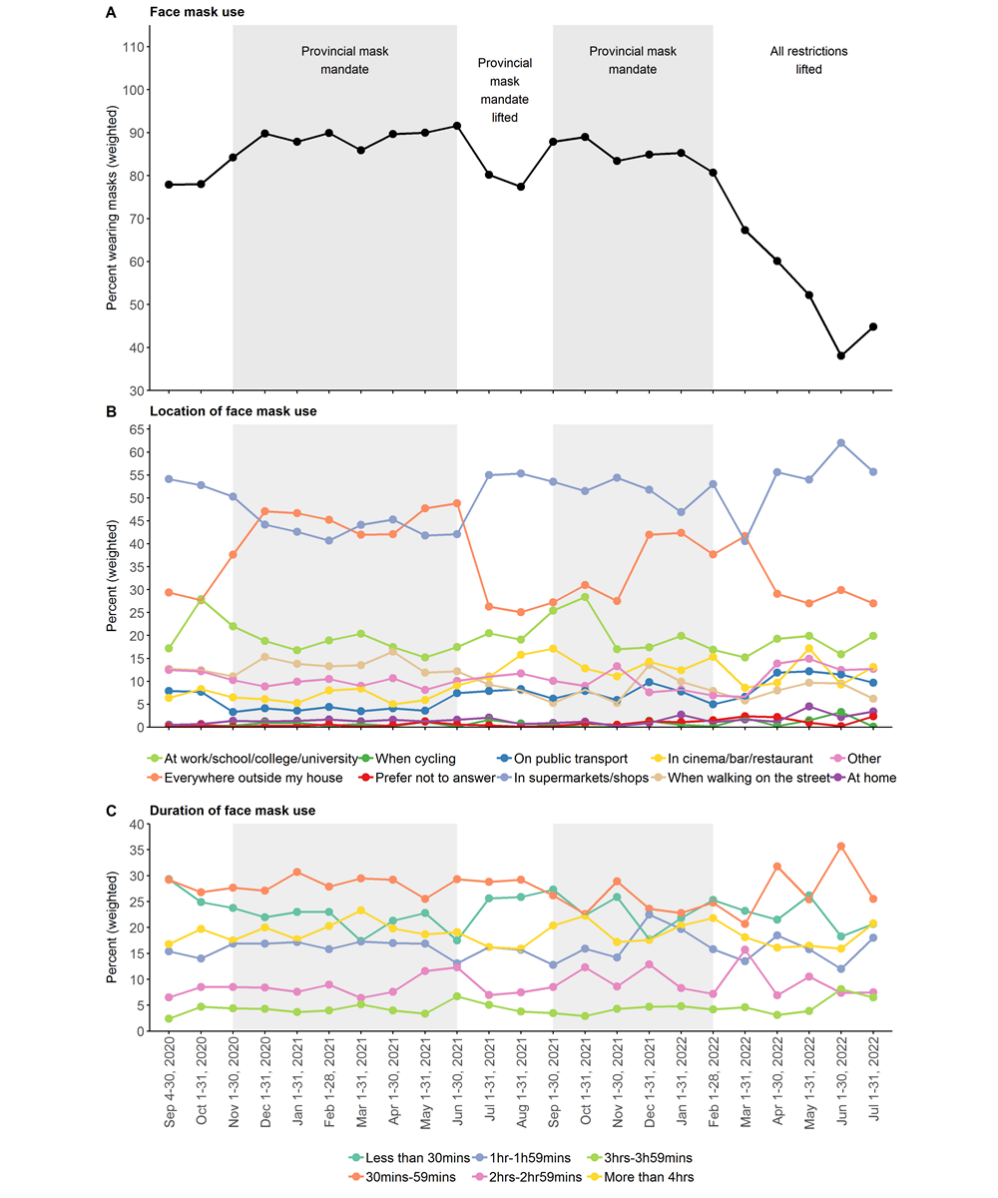
Face mask usage patterns among British Columbia COVID-19 Population Mixing Patterns (BC-Mix) survey respondents by month, from September 2020 to July 2022. (A) Face mask use rates. (B) Location of face mask use: percentages calculated independently for each option provided. (C) Duration of face mask use. Shaded gray region: provincial mask mandate in effect.

Between September 2020 and July 2022, 81.9% of survey respondents reported wearing a face mask outside their homes the day before completing the survey (face mask use: yes, n=36,716; no, n=7585 [*prefer not to answer*, n=155 + *no*, n=7430]). Face mask usage rates were approximately 78% between September and October 2020 when face masks and coverings were required in major retail locations in BC but not provincially mandated. Face mask usage rates increased following the introduction of the provincial mask mandate in November 2020 and remained at or above 84% each month thereafter until the mandate was first lifted in July 2021 (Figure 1A). After a 2-month decline in face mask usage rates to pre–provincial mandate levels, usage rates rebounded to 87.9% in September 2021 once the provincial mask mandate was reinstated at the end of August 2021. As before, face mask usage rates remained above 80% when the provincial mandate was in effect until March 2022, when the mandate was lifted a final time as an important step in the winding down of control measures across the province. Face mask usage rates fell rapidly soon afterward, reaching 38.1% in June 2022.

Face mask usage patterns were generally consistent over the 23-month period (Figure 1A). Masks were primarily worn in supermarkets and shops (48.2% of face mask users) and everywhere outside the house (38.2% of face mask users; Figure 1B and Figure S1A in the Multimedia Appendix 1).The proportion of people who wore face masks everywhere outside their homes remained at or above 25%, with face mask users being more likely to have worn face masks everywhere outside their homes when provincial mask mandates were in effect. Regardless of time period, most face mask users reported wearing their mask for 59 minutes or less (Figure 1C and Figure S1B in the Multimedia Appendix 1).

**Figure 2:**
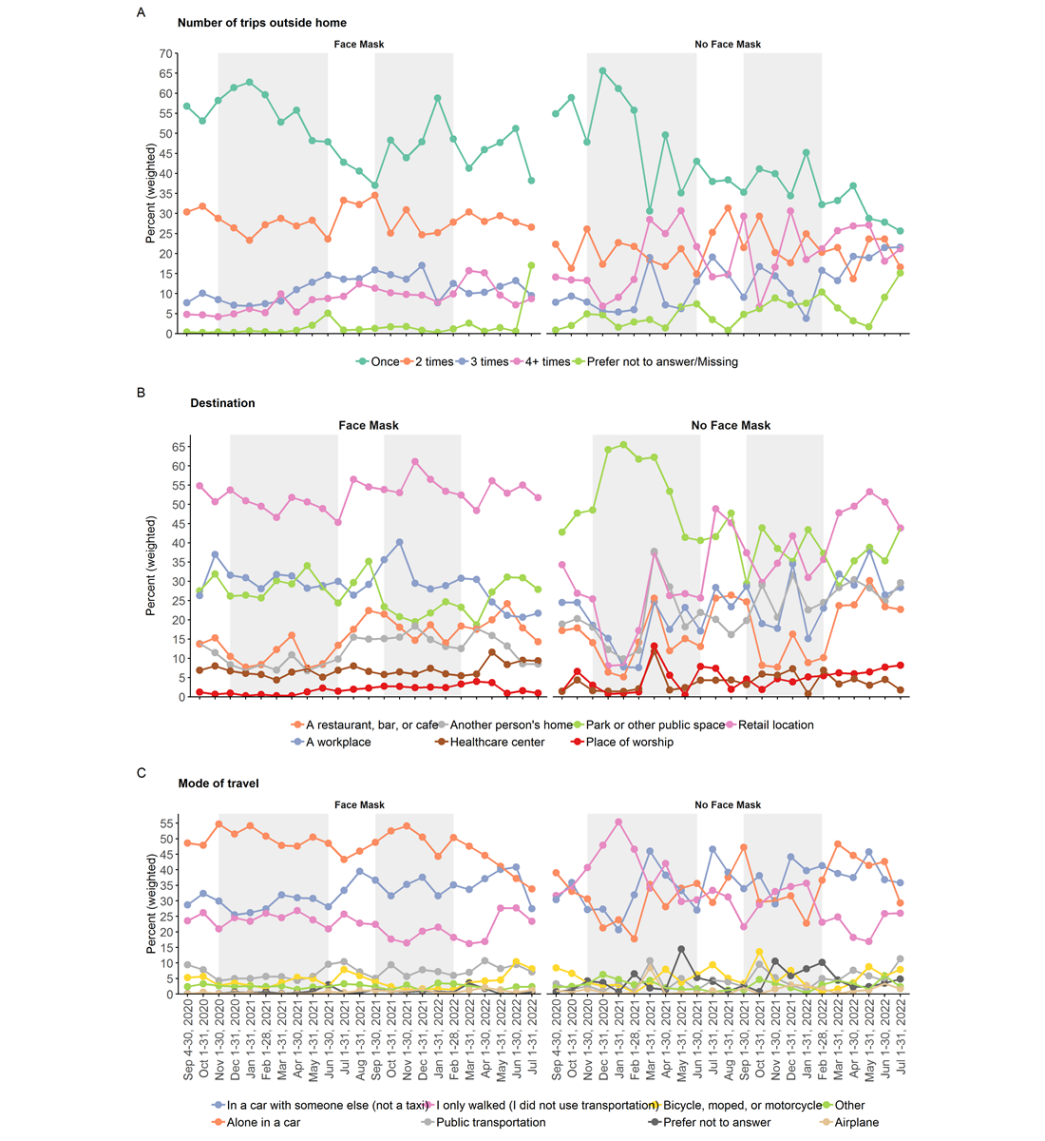
Travel patterns of British Columbia COVID-19 Population Mixing Patterns (BC-Mix) survey respondents by face mask use by month, from September 2020 to July 2022. (A) Number of trips taken outside the home. (B) Destination: percentages calculated independently for each option provided. (C) Mode of travel: percentages calculated independently for each option provided. Shaded gray region: provincial mask mandate in effect.

Travel patterns were distinct between users and nonusers of face masks between September 2020 and July 2022. The majority of face mask users (53.2%) and nonusers (43.5%) left home only once the previous day, although nonusers of face masks were at least twice as likely to leave home 4 times or more (18.2% vs 7.3%; Figure S2A in the Multimedia Appendix 1 and Figure 2A). Retail locations including grocery stores, pharmacies, and liquor stores were the most frequented destinations for face mask users throughout the 23-month period (≥45%; Figure S2A in the Multimedia Appendix 1 and Figure 2A). Among nonusers of face masks, however, parks or public spaces were the most common destinations visited prior to June 2021, which was gradually surpassed by retail locations after the lifting of provincial mask mandates (Figure 2B). Face mask users mostly travelled alone in a car, although the mode of travel was more heterogeneous among nonusers of face masks during the 23-month period (Figure 2C and Figure S2C in the Multimedia Appendix 1). Statistically significant differences were observed in the distributions of face mask users and nonusers by sex, where larger proportions of females than males opted for wearing masks (85% vs 79%; *P*<.001; Table 1). This difference was more evident when survey respondents were grouped by destination (Table 2, Table 3). In terms of face mask group composition, males formed a large majority of people who opted out of wearing face masks when visiting retail locations (74.9%); restaurants, bars, or cafés (76.1%); or workplaces (78%).

**Table 2:** Proportion of British Columbia COVID-19 Population Mixing Patterns (BC-Mix) survey respondents by destination^a^ and face mask use (yes or no), stratified “across” age groups and sex, from September 2020 to July 2022.

Characteristic		Retail location (n=21,732)		Restaurant, bar, or café (n=5183)		Workplace (n=10,226)		Park or other public space (n=14,577)	
		Yes (n=19,761), %^b^	No (n=1971), %^b^	Yes (n=4239), %^b^	No (n=944), %^b^	Yes (n=9120), %^b^	No (n=1106), %^b^	Yes (n=10,735), %^b^	No (n=3842), %^b^
**Age group (years)**									
	18-24	6.4	9.5	10.4	13	11.8	14.6	7.4	7.7
	25-34	16.8	20	21.7	20.1	24.8	22.1	21.8	21.3
	35-44	14.6	18.2	15.8	17.2	19.2	18.1	18	15.3
	45-54	19.3	19.4	18.1	20.4	22.9	24.9	16.9	18.2
	55-64	17.5	15.2	14.4	12.7	15.9	14	15.1	15.9
	65-74	19	13.8	14.6	12	4.7	5.1	16	16.8
	≥75	6.3	4	4.9	4.4	0.6	1.1	4.8	4.8
**Sex**									
	Female	48.1	25.1	42.3	23.9	46.7	22	52.7	43.9
	Male	51.9	74.9	57.7	76.1	53.3	78	47.3	56.1

**^a^**Selected individually—percentages were calculated for each option provided.

^b^Weighted percentages.

**Table 3:** Proportion of British Columbia COVID-19 Population Mixing Patterns (BC-Mix) survey respondents by destination^a^, stratified as face mask users and nonusers “within” each age group and sex, from September 2020 to July 2022.

Characteristic		Retail location (n=21,732)		Restaurant, bar, or café (n=5183)		Workplace (n=10,226)		Park or other public space (n=14,577)	
		User (n=19,761), %^b^	Nonuser (n=1971), %^b^	User (n=4239), %^b^	Nonuser (n=944), %^b^	User (n=9120), %^b^	Nonuser (n=1106), %^b^	User (n=10,735), %^b^	Nonuser (n=3842), %^b^
**Age group (years)**									
	18-24	82.3	17.7	72.8	27.2	82.7	17.3	72.5	27.5
	25-34	85.3	14.7	78.3	21.7	86.9	13.1	73.5	26.5
	35-44	84.6	15.4	75.4	24.6	86.3	13.7	76.2	23.8
	45-54	87.2	12.8	74.9	25.1	84.5	15.5	71.6	28.4
	55-64	88.8	11.2	79.1	20.9	87	13	72.1	27.9
	65-74	90.5	9.5	80.2	19.8	84.5	15.5	72.1	27.9
	≥75	91.5	8.5	78.5	21.5	76.6	23.4	73.2	26.8
**Sex**									
	Female	92.9	7.1	85.5	14.5	92.6	7.4	76.5	23.5
	Male	82.6	17.4	71.7	28.3	80.2	19.8	69.6	30.4

**^a^**Selected individually—percentages were calculated for each option provided.

^b^Weighted percentages.

There were small differences in the demographic distributions of people who reported wearing or not wearing face masks in the presence and absence of the provincial mask mandate (Table S3 in the Multimedia Appendix 1). However, the shift toward decreased face mask use when the provincial mask mandate was not in effect was evident across people of all sexes, age groups, and ethnicities, regardless of their level of education, occupation, or employment status.

### Factors Associated with Face Mask Use

**Figure 3:**
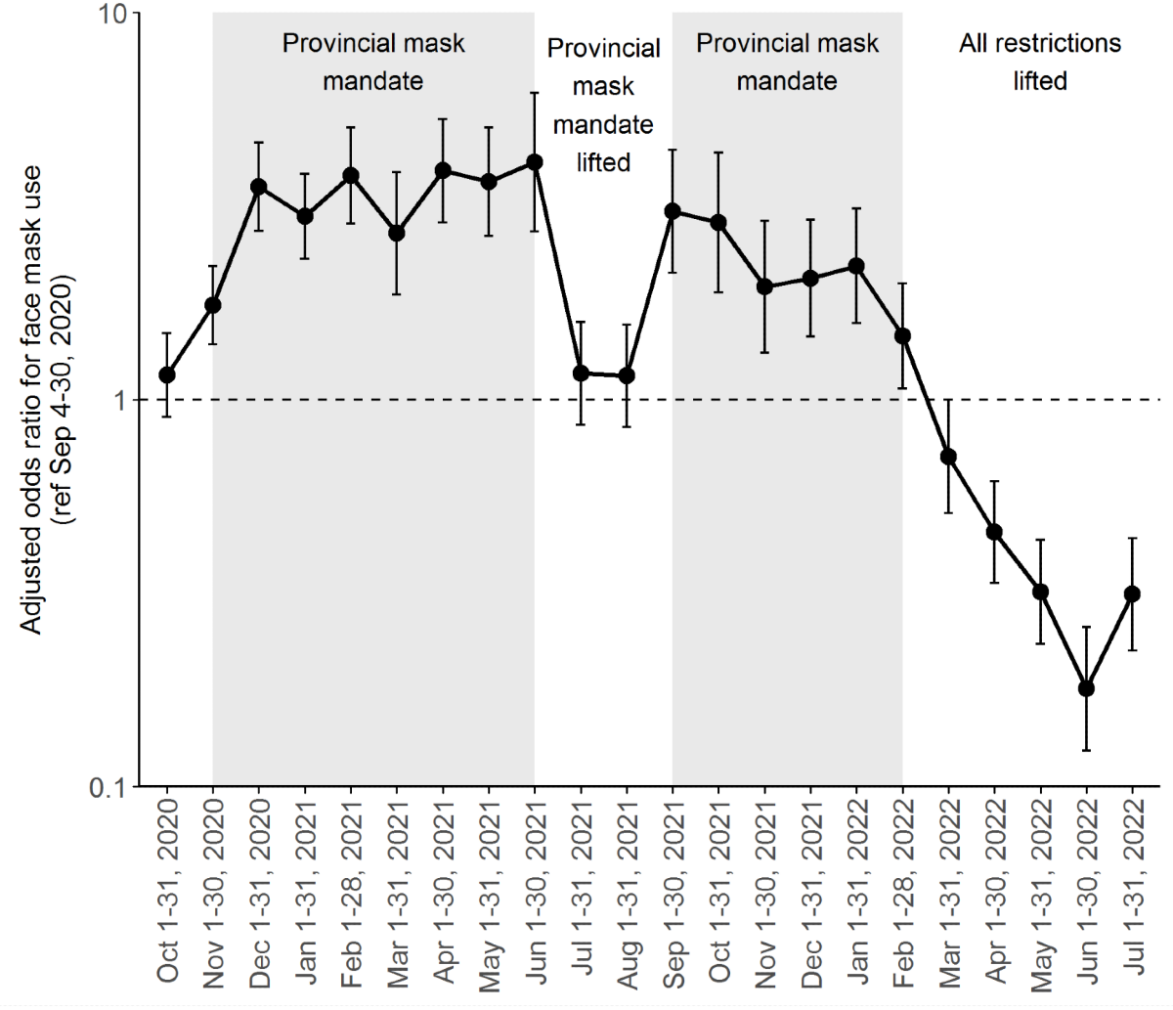
Odds ratios for face mask use among British Columbia COVID-19 Population Mixing Patterns (BC-Mix) survey respondents by time period, from September 2020 to July 2022.

In a multivariable logistic regression model, the odds of wearing a face mask were statistically significantly greater during the months when the mask mandate was in effect (all *P*<.05; Figure 3 and Table S2 in the Multimedia Appendix 1). The destination and mode of travel were associated with face mask use, where people going to retail locations, including grocery stores, pharmacies, and liquor stores, had greater odds of wearing face masks than those going to parks or other public spaces (adjusted odds ratio [aOR] 14.23, 95% CI 11.69-17.31), as did persons travelling alone in a car (aOR 2.15, 95% CI 1.86-2.50) or in a car with someone else (aOR 1.59, 95% CI 1.36-1.86) relative to those who only walked to their destinations (Table S2 in the Multimedia Appendix 1). Compared to people who were not part of a visible minority group, Chinese people (aOR 2.02, 95% CI 1.54-2.65), South Asian people (aOR 1.80, 95% CI 1.27-2.56), and others who were part of a visible minority group (aOR 1.89, 95% CI 1.49-2.40) had greater odds of wearing face masks. The odds of face mask use were also greater among females, people aged ≥65 years, and people living in the more populous health regions.

The impact of the provincial mask mandates was even more evident when examined directly, with 3.68 times greater odds of face mask use when the provincial mask mandate was in effect (aOR 3.68, 95% CI 3.33-4.05; Table S4 in the Multimedia Appendix 1). The odds ratios for face mask use increased more than 2-fold among people whose destinations were indoor public spaces such as restaurants, bars, or cafés (aOR 7.35, 95% CI 4.23-12.78 vs aOR 2.81, 95% CI 1.50-5.26) or retail locations (aOR 19.94, 95% CI 14.86-26.77 vs aOR 7.71, 95% CI 5.68-10.46) with the mask mandate versus without. Slight shifts in odds ratios were also noted by mode of travel in the presence versus the absence of a provincial mask mandate.

## Discussion

### Principal Findings

Prior to the availability and high coverage of COVID-19 vaccines, masks and other nonpharmaceutical interventions were mainstays for preventing infection and reducing disease transmission, with the ultimate goal of reducing the impact of the COVID-19 pandemic on population health. Face mask use was mandated in certain settings across many countries to reduce SARS-CoV-2 transmission. Establishing the factors associated with and the patterns of face mask use, with or without mask mandates, is necessary to assess the impact of mask mandates and to inform health communication strategies and decision-making by public health leadership. In this study, based on survey responses from a voluntary sample of BC residents conducted between September 2020 and July 2022, 81.9% of respondents reported wearing a face mask during outings. Over the 23-month period, face masks were mostly worn for less than an hour, being primarily used in supermarkets and shops; at workplaces; and in schools, colleges, or universities. In a multivariable logistic regression model, factors associated with face mask use included age, sex, ethnicity, time period, destination, and mode of travel. Face mask usage rates were sustained by in-store mandates in the fall of 2020 and further boosted by the provincial mandates. The odds of face mask use increased 3-fold when the provincial mask mandate was in effect. These findings highlight the role of mask mandates in facilitating high levels of face mask use at the population level.

Studies based in the United States have shown an increased likelihood of face mask use in indoor public spaces, such as grocery stores, compared to outdoor public spaces, such as parks or beaches [43,44]. Similarly, retail locations and workplaces were among the major destinations associated with face mask use in BC, whereas parks were favored among people who opted against wearing masks. This finding was to be expected, as in-store and regional mask mandates were in effect in most of these locations for the majority of the study period. Differences in face mask use at retail locations have been reported in the United States, where females [45], individuals aged ≥65 years [45], non-Hispanic Black and Hispanic/Latino persons [46], and people shopping in urban or suburban locations [45] were among the most likely to have worn face masks prior to the introduction of mask mandates or recommendations. Similarly, females and people aged ≥55 years had greater odds of adopting face masks across Canada [28]. This was congruent with our findings in BC, where statistically significant differences in face mask use were noted by age and sex—males were more likely to be nonusers of face masks, especially in commonly frequented settings.

Our findings, and those of others, highlight the impact of mask mandates in promoting face mask use during the COVID-19 pandemic [45,47]. Province-wide in-store mask mandates sustained face mask usage rates in BC at approximately 80%, both before the introduction of the provincial mask mandate and during the 2-month period when the mandate was first lifted. Moreover, face mask usage rates were at or above 84% when provincial mask mandates were in effect, similar to findings in the United States and Australia [45,47]. The greatest impact of the mask mandate on the odds of face mask use was seen at key locations such as workplaces, restaurants, bars, cafes, grocery stores, liquor stores, and pharmacies. Once removed, alongside other control measures, face mask usage rates declined 2-fold to 38%, possibly reflecting baseline midpandemic mask usage rates in the absence of mask mandates.

### Limitations

Study findings should be interpreted with the following limitations in mind. Data collection began after face masks were made mandatory on public transit and in many retail locations in BC; thus, we were unable to fully quantify premandate willingness to voluntarily wear face masks at these locations. Nevertheless, our data does contribute to the body of knowledge about (un)willingness to wear face masks in the face of regional or in-store mandates, as a sizeable proportion of respondents fell into this category. Our findings are also subject to selection bias, as survey respondents were recruited mainly on social media platforms (Instagram, Facebook, YouTube, and Twitter) and participated on a voluntary basis. Thus, persons who did not use these social media platforms would not have been able to participate without referral. Furthermore, we were not able to quantify nonparticipation as recruitment was done in a passive manner. In addition, we did not assess type of face masks used, which may have provided additional insights into the characteristics and behaviors of survey respondents. Nevertheless, our study provides valuable insight into the dynamics of face mask use during the COVID-19 pandemic.

### Conclusions

Various studies have shown the association between face mask use and declines in SARS-CoV-2 transmission [21-23]. Thus, in the absence of vaccines for disease prevention and therapeutics for the treatment and prevention of severe disease, mask mandates were introduced during the COVID-19 pandemic to limit the spread of the disease and to reduce its impact on society. We found a pattern of high mask usage rates with retail location and public transit mask mandates in BC, which was further enhanced by the provincial mask mandate. These findings demonstrate the utility of mask mandates in sustaining high rates of face mask use during the COVID-19 pandemic and provide concrete evidence for their use in regions with low vaccination rates and recurrent surges in COVID-19 cases and in the event of future respiratory virus-driven pandemics or severe respiratory disease outbreaks. Lessons learned from the COVID-19 pandemic do suggest, however, that mask mandate imposition should require a sound ethical analysis beforehand to ensure that the benefits achieved with their use outweigh the harms related to infringement on individual choices.

## Appendix

Multimedia Appendix 1 Supplementary materials.

## Abbreviations

aOR: adjusted odds ratio
BC: British Columbia
BC-Mix: British Columbia COVID-19 Population Mixing Patterns

## Data Availability

Data are available upon reasonable request.

## References

[1] Güner Rahmet, Hasanoğlu I, Aktaş F (2020). COVID-19: prevention and control measures in community. Turk J Med Sci.

[2] (2020). Listings of WHO's response to COVID-19. World Health Organization.

[3] Leung Nancy H L, Chu Daniel K W, Shiu Eunice Y C, Chan Kwok-Hung, McDevitt James J, Hau Benien J P, Yen Hui-Ling, Li Yuguo, Ip Dennis K M, Peiris J S Malik, Seto Wing-Hong, Leung Gabriel M, Milton Donald K, Cowling Benjamin J (2020). Respiratory virus shedding in exhaled breath and efficacy of face masks. Nat Med.

[4] Alsved M, Matamis A, Bohlin R, Richter M, Bengtsson P, Fraenkel C, Medstrand P, Löndahl J (2020). Exhaled respiratory particles during singing and talking. Aerosol Sci Technol.

[5] Gandhi M, Marr LC (2021). Uniting infectious disease and physical science principles on the importance of face masks for COVID-19. Med (N Y).

[6] Gandhi M, Beyrer C, Goosby E (2020). Masks do more than protect others during COVID-19: reducing the inoculum of SARS-CoV-2 to protect the wearer. J Gen Intern Med.

[7] (2020). Science brief: community use of cloth masks to control the spread of SARS-CoV-2. Centers for Disease Control and Prevention.

[8] Hendrix MJ, Walde C, Findley K, Trotman R (2020). Absence of apparent transmission of SARS-CoV-2 from two stylists after exposure at a hair salon with a universal face covering policy - Springfield, Missouri, May 2020. MMWR Morb Mortal Wkly Rep.

[9] Lyu W, Wehby GL (2020). Community use of face masks and COVID-19: evidence from a natural experiment of state mandates in the US. Health Aff (Millwood).

[10] Howard J, Huang A, Li Z, Tufekci Z, Zdimal V, van der Westhuizen H, von Delft A, Price A, Fridman L, Tang L, Tang V, Watson GL, Bax CE, Shaikh R, Questier F, Hernandez D, Chu LF, Ramirez CM, Rimoin AW (2021). An evidence review of face masks against COVID-19. Proc Natl Acad Sci U S A.

[11] Guy GP, Lee FC, Sunshine G, McCord R, Howard-Williams M, Kompaniyets L, Dunphy C, Gakh M, Weber R, Sauber-Schatz E, Omura JD, Massetti GM, CDC COVID-19 Response Team‚ Mitigation Policy Analysis Unit, CDC Public Health Law Program (2021). Association of state-issued mask mandates and allowing on-premises restaurant dining with county-level COVID-19 case and death growth rates - United States, March 1-December 31, 2020. MMWR Morb Mortal Wkly Rep.

[12] Li Y, Liang M, Gao L, Ayaz Ahmed M, Uy JP, Cheng C, Zhou Q, Sun C (2021). Face masks to prevent transmission of COVID-19: a systematic review and meta-analysis. Am J Infect Control.

[13] (2021). Mask mandates by nation: most still await a breath of fresh air. Bloomberg News.

[14] Feng S, Shen C, Xia N, Song W, Fan M, Cowling BJ (2020). Rational use of face masks in the COVID-19 pandemic. Lancet Respir Med.

[15] Prasad R (2020). Coronavirus: why is there a US backlash to masks?. BBC News.

[16] Jarry J (2020). Why some people choose not to wear a mask. McGill Office for Science and Society.

[17] (2020). Mandating masks will remain the job of businesses and services, Dr. Bonnie Henry says. CBC News.

[18] Taylor S, Asmundson GJG (2021). Negative attitudes about facemasks during the COVID-19 pandemic: the dual importance of perceived ineffectiveness and psychological reactance. PLoS One.

[19] Haraf R, Faghy M, Carlin B, Josephson R (2021). The physiological impact of masking is insignificant and should not preclude routine use during daily activities, exercise, and rehabilitation. J Cardiopulm Rehabil Prev.

[20] Howard MC (2020). Understanding face mask use to prevent coronavirus and other illnesses: development of a multidimensional face mask perceptions scale. Br J Health Psychol.

[21] Krishnamachari B, Morris A, Zastrow D, Dsida A, Harper B, Santella A (2021). The role of mask mandates, stay at home orders and school closure in curbing the COVID-19 pandemic prior to vaccination. Am J Infect Control.

[22] Van Dyke ME, Rogers TM, Pevzner E, Satterwhite CL, Shah HB, Beckman WJ, Ahmed F, Hunt DC, Rule J (2020). Trends in county-level COVID-19 incidence in counties with and without a mask mandate - Kansas, June 1-August 23, 2020. MMWR Morb Mortal Wkly Rep.

[23] Mitze T, Kosfeld R, Rode J, Wälde Klaus (2020). Face masks considerably reduce COVID-19 cases in Germany. Proc Natl Acad Sci U S A.

[24] Peeples L (2021). What the science says about lifting mask mandates. Nature.

[25] Kresge N (2022). Europe Can’t Shake Off Covid as Variant Fuels Summer Spike. Bloomberg.

[26] (2022). French authorities 'encourage' mask use as COVID cases surge. EuroNews.

[27] St. George D (2022). School mask mandates return as latest coronavirus variants surge. The Washington Post.

[28] Lang R, Atabati O, Oxoby RJ, Mourali M, Shaffer B, Sheikh H, Fullerton MM, Tang T, Leigh JP, Manns BJ, Marshall DA, Ivers NM, Ratzan SC, Hu J, Benham JL (2021). Characterization of non-adopters of COVID-19 non-pharmaceutical interventions through a national cross-sectional survey to assess attitudes and behaviours. Sci Rep.

[29] Benham JL, Lang R, Kovacs Burns K, MacKean G, Léveillé Tova, McCormack B, Sheikh H, Fullerton MM, Tang T, Boucher J, Constantinescu C, Mourali M, Oxoby RJ, Manns BJ, Hu J, Marshall DA (2021). Attitudes, current behaviours and barriers to public health measures that reduce COVID-19 transmission: a qualitative study to inform public health messaging. PLoS One.

[30] Government of British Columbia (2021). COVID-19 Information and Resources. EmergencyInfoBC.

[31] (2021). Ministerial order no. M012. British Columbia Minister of Public Safety and Solicitor General.

[32] Weichel A (2020). B.C. announces mask mandate, temporary social lockdown for entire province. CTV News.

[33] (2020). Where do you need to wear a mask in B.C.? Here are some places where they are mandatory. CBC News.

[34] Ross A (2020). Face masks will be mandatory on public transit across much of B.C. CBC News.

[35] Zussman R (2021). B.C. drops mandatory indoor mask mandate starting July 1. Global News.

[36] (2021). Mask mandate to reduce transmission, protect people in public spaces. British Columbia Ministry of Health.

[37] (2021). Indoor mask mandate extended. British Columbia Ministry of Health.

[38] (2022). B.C. takes next step in balanced plan to lift COVID-19 restrictions. Government of British Columbia.

[39] Adu PA, Binka M, Mahmood B, Jeong D, Buller-Taylor T, Damascene MJ, Iyaniwura S, Ringa N, Velásquez García Héctor A, Wong S, Yu A, Bartlett S, Wilton J, Irvine MA, Otterstatter M, Janjua NZ (2022). Cohort profile: the British Columbia COVID-19 Population Mixing Patterns Survey (BC-Mix). BMJ Open.

[40] Bethlehem J (2009). Applied survey methods: A statistical perspective.

[41] R Core Team R: a language and environment for statistical computing. The R Foundation for Statistical Computing.

[42] (2021). What is SAS?. SAS Institute Inc.

[43] Knotek ES II, Schoenle RS, Dietrich AM, Müller GJ, Myrseth KOR, Weber M (2020). Consumers and COVID-19: survey results on mask-wearing behaviors and beliefs. Federal Reserve Bank of Cleveland, Economic Commentary.

[44] Schoeni RF, Wiemers EE, Seltzer JA, Langa KM (2021). Association between risk factors for complications from COVID-19, perceived chances of infection and complications, and protective behavior in the US. JAMA Netw Open.

[45] Haischer MH, Beilfuss R, Hart MR, Opielinski L, Wrucke D, Zirgaitis G, Uhrich TD, Hunter SK (2020). Who is wearing a mask? gender-, age-, and location-related differences during the COVID-19 pandemic. PLoS One.

[46] Fisher KA, Barile JP, Guerin RJ, Vanden Esschert KL, Jeffers A, Tian LH, Garcia-Williams A, Gurbaxani B, Thompson WW, Prue CE (2020). Factors associated with cloth face covering use among adults during the COVID-19 pandemic - United States, April and May 2020. MMWR Morb Mortal Wkly Rep.

[47] Scott N, Saul A, Spelman T, Stoove M, Pedrana A, Saeri A, Grundy E, Smith L, Toole M, McIntyre CR, Crabb BS, Hellard M (2021). The introduction of a mandatory mask policy was associated with significantly reduced COVID-19 cases in a major metropolitan city. PLoS One.

