# Supplementary material for: The Impact of Mask Mandates on Face Mask Use During the COVID-19 Pandemic: Longitudinal Survey Study
Source: JMIR Public Health Surveill. 2023 Jan 11;9:e42616. doi: 10.2196/42616 (PMC9838719; doi:10.2196/42616)

**Multimedia Appendix 1**

**Title:** The Impact of Mask Mandates on Face Mask Use During the COVID-19 Pandemic: Longitudinal Survey Study

**Authors:** Mawuena Binka,^1^ Prince Asumadu Adu,^1,2^ Dahn Jeong,^1,2^ Nirma Khatri Vadlamudi,^3^ Héctor Alexander Velásquez García,^1,2^ Bushra Mahmood,^1^ Terri Buller-Taylor,^1^ Michael Otterstatter,^1,2^ Naveed Zafar Janjua^1,2^

**Author affiliations:**

1. British Columbia Centre for Disease Control, Vancouver, British Columbia, Canada
2. School of Population and Public Health, University of British Columbia, Vancouver, Canada
3. Faculty of Pharmaceutical Sciences, University of British Columbia, Vancouver, Canada

**Table of contents:**

**Table S1.** Relevant British Columbia COVID-19 Population Mixing Patterns (BC-Mix) survey questions, September 2020-July 2022

**Table S2.** Odds ratios for face mask usage among British Columbia COVID-19 Population Mixing Patterns (BC-Mix) survey respondents from a multivariable logistic regression model, September 2020-July 2022

**Table S3.** Characteristics of British Columbia COVID-19 Population Mixing Patterns (BC-Mix) survey respondents by face mask usage in the presence and absence of the provincial mask mandate, September 2020-July 2022

**Table S4.** Odds ratios for face mask usage among British Columbia COVID-19 Population Mixing Patterns (BC-Mix) survey respondents in the presence or absence of the provincial mask mandate, September 2020-July 2022

**Figure S1.** Face mask usage patterns among BC-Mix survey respondents, September 2020-July 2022. (A) Location of face mask use: Percentages calculated independently for each option provided. (B) Duration of face mask use.

**Figure S2.** Travel patterns of BC-Mix survey respondents by face mask use, September 2020-July 2022. (A) Number of trips taken outside the home. (B) Destination: Percentages calculated independently for each option provided. (C) Mode of travel: Percentages calculated independently for each option provided.

**Table S1.** Relevant British Columbia COVID-19 Population Mixing Patterns (BC-Mix) survey questions, September 2020-July 2022

| **Variable** | **Question/definition** | **Response categories** | **Derived categories** |
| --- | --- | --- | --- |
| Age | What is your age? | 18-24 | 18-24 years |
|  |  | 25-34 | 25-34 years |
|  |  | 35-44 | 35-44 years |
|  |  | 45-54 | 45-54 years |
|  |  | 55-64 | 55-64 years |
|  |  | 65-74 | 65-74 years |
|  |  | 75 or greater | 75+ years |
|  |  | Prefer not to answer | Prefer not to answer |
| Sex | What is your sex? | Male | Male |
|  |  | Female | Female |
|  |  | Other | - |
|  |  | Prefer not to answer | - |
| Ethnicity | Do you consider yourself to be (check all that apply) | First Nations | Other ethnicity |
|  |  | Métis | Other ethnicity |
|  |  | Inuit | Other ethnicity |
|  |  | White (European descent) | Not a visible minority |
|  |  | Chinese | Chinese |
|  |  | South Asian (e.g. East Indian, Pakistani, Sri Lankan) | South Asian |
|  |  | Black (e.g. African or Caribbean) | Other visible minority |
|  |  | Filipino | Other visible minority |
|  |  | Latin American/Hispanic | Other visible minority |
|  |  | Southeast Asian (e.g. Vietnamese, Cambodian, Malaysian, Laotian) | Other visible minority |
|  |  | Arab | Other visible minority |
|  |  | West Asian (e.g. Iranian, Afghan) | Other visible minority |
|  |  | Korean | Other visible minority |
|  |  | Japanese | Other visible minority |
|  |  | Other, prefer to self describe | Other ethnicity |
|  |  | Prefer not to answer | Prefer not to answer |
|  |  |  | Missing |
| Education | What is the highest level of school you have completed or the highest degree you have received? | Less than high school degree | Below High School |
|  |  | High school graduate (high school diploma or equivalent including GED) | Below Bachelor's degree |
|  |  | Some college/university but no degree | Below Bachelor's degree |
|  |  | Associate degree or diploma in college/university (2-year) | Below Bachelor's degree |
|  |  | Bachelor's degree in college (4-year) | University degree |
|  |  | Master's degree | University degree |
|  |  | Doctoral Degree | University degree |
|  |  | Professional degree (e.g. JD, MD) | University degree |
|  |  | Prefer not to answer | Prefer not to answer/Missing |
|  |  |  | Prefer not to answer/Missing |
| Employment status | What is your current employment status? | Employed full-time (30 hours or more/week) | Employed full-time |
|  |  | Employed part-time (less than 30 hours/week) | Employed part-time |
|  |  | Self-employed | Self-employed |
|  |  | Unemployed but looking for a job | Unemployed |
|  |  | Unemployed and not looking for a job | Unemployed |
|  |  | Full-time parent, homemaker | Full-time parent, homemaker |
|  |  | Retired | Retired |
|  |  | Student/Pupil | Student/Pupil |
|  |  | Long-term sick or disabled | Long-term sick/disabled |
|  |  | Prefer not to answer | Prefer not to answer/Missing |
|  |  |  | Prefer not to answer/Missing |
| Occupation | As of March 2020, what occupation or industry have you most often worked in? | I do not work | Do not work |
|  |  | Business, finance and administation occupations | Non-essential workers |
|  |  | Health occupations (e.g., medical, social work, psychology) | Essential workers |
|  |  | Management occupations | Non-essential workers |
|  |  | Natural and applied sciences and related occupations | Non-essential workers |
|  |  | Natural resources, agriculture and related production occupations | Essential workers |
|  |  | Occupations in art, culture, recreation and sport | Non-essential workers |
|  |  | Occupations in education, law and social, community and government services | Non-essential workers |
|  |  | Occupations in manufacturing and utilities | Essential workers |
|  |  | Sales and service occupations | Essential workers |
|  |  | Trades, transport and equipment operators and related occupations | Essential workers |
|  |  | Other (prefer to self describe) | Others |
|  |  | Prefer not to answer | Prefer not to answer/Missing |
|  |  |  | Prefer not to answer/Missing |
| Number of trips outside home | How many times did you leave your home (or property, apartment) yesterday? | Did not leave | - |
|  |  | Once | Once |
|  |  | 2 times | 2 times |
|  |  | 3 times | 3 times |
|  |  | 4 times | 4+ times |
|  |  | 5 times | 4+ times |
|  |  | 6 or more times | 4+ times |
|  |  | Prefer not to answer | Prefer not to answer/Missing |
|  |  |  | Prefer not to answer/Missing |
| Destination^a^ | Where did you go when you left your home? (Check all that apply) | Another person's home | Another person's home |
|  |  | A workplace | A workplace |
|  |  | A hospital, doctor's office, or other healthcare center | Other destinations |
|  |  | Retail including grocery store, pharmacy, liquor store | Retail including grocery store, pharmacy, liquor store |
|  |  | Church, Temple, or other place of worship | Other destinations |
|  |  | A shared space in my building or residential compound | Other destinations |
|  |  | A restaurant, bar, or café | A restaurant, bar, or café |
|  |  | Small event < 10 people | Other destinations |
|  |  | Medium size event 10-50 people | Other destinations |
|  |  | Large event (music concert, sports game, movie, etc.) | Other destinations |
|  |  | Park or other public space (including walking along a sidewalk) | Park or other public space |
|  |  | **Multiple selections* | Multiple destinations |
|  |  | Other | Other destinations |
|  |  | Prefer not to answer | Prefer not to answer/Missing |
|  |  |  | Prefer not to answer/Missing |
| Mode of travel^a^ | How did you travel when you left your home? (Check all that apply) | I only walked (I did not use other transportation) | I only walked (I did not use transportation) |
|  |  | Bicycle, moped, or motorcycle | Bicycle, moped, or motorcycle |
|  |  | Airplane | Other/Prefer not to answer |
|  |  | Public transportation (e.g. bus, train, subway, tram, airplane etc) | Other/Prefer not to answer |
|  |  | Alone in a car | Alone in a car |
|  |  | In a car with someone else (not a taxi) | In a car with someone else (not a taxi) |
|  |  | **Multiple selections* | Multiple modes of travel |
|  |  | Other (please specify) | Other/Prefer not to answer |
|  |  | Prefer not to answer | Other/Prefer not to answer |
| Distance traveled | What is the farthest distance that you went from your home yesterday? | Less than 1 kilometre | Less than 1 km |
|  |  | 1 to 10 kilometres | 1 to 10 km |
|  |  | 10 to 100 kilometres | 10 to 100 km |
|  |  | More than 100 kilometres | More than 100 km |
|  |  | Prefer not to answer | Prefer not to answer/Missing |
|  |  |  | Prefer not to answer/Missing |
| Health Authority | This is a derived variable using respondents’ postal code. | Postal code | Interior Health |
|  |  |  | Fraser Health |
|  |  |  | Vancouver Coastal Health |
|  |  |  | Vancouver Island Health |
|  |  |  | Northern Health |
|  |  |  | Missing |
| Time period | This variable is derived from the survey recorded date | Recorded date | Month, Year |
| ^a^Used both as a derived variable in logistic regression models, and has options split into individual variables in descriptive analyses. | | | |

**Table S2.** Odds ratios for face mask usage among British Columbia COVID-19 Population Mixing Patterns (BC-Mix) survey respondents from a multivariable logistic regression model, September 2020-July 2022.

|  | **Face Mask Use: Yes^a^** | **Face Mask Use: No^a^** | **Face Mask Use: Yes^a^** | **Face Mask Use: No^a^** | **Face Mask Use: Yes** | **Face Mask Use: No** |  |  |
| --- | --- | --- | --- | --- | --- | --- | --- | --- |
|  | **n (unweighted)** | **n (unweighted)** | **column % (weighted)** | **column % (weighted)** | **row % (weighted)** | **row % (weighted)** | **Adjusted Odds Ratio (95% CI)** | ***P*-value** |
| **Age** |  |  |  |  |  |  |  |  |
| 18-24 years | 1095 | 239 | 8.4 | 9.6 | 79.8 | 20.2 | 0.75 (0.56-1.01) | .06 |
| 25-34 years | 3459 | 696 | 18.6 | 19.6 | 81.1 | 18.9 | **0.64 (0.50-0.81)** | **<.001** |
| 35-44 years | 5157 | 1066 | 15.9 | 16.2 | 81.6 | 18.4 | **0.71 (0.57-0.89)** | **.003** |
| 45-54 years | 6409 | 1159 | 18.7 | 18.2 | 82.3 | 17.7 | **0.71 (0.57-0.89)** | **.002** |
| 55-64 years | 9149 | 1969 | 17.0 | 16.1 | 82.7 | 17.3 | **0.68 (0.55-0.84)** | **<.001** |
| 65-74 years | 8817 | 1886 | 16.1 | 15.5 | 82.4 | 17.6 | 0.85 (0.69-1.03) | .10 |
| 75+ years | 2630 | 570 | 5.2 | 4.8 | 83.0 | 17.0 | Reference | - |
| **Sex** |  |  |  |  |  |  |  |  |
| Female | 29833 | 5737 | 49.5 | 39.4 | 85.0 | 15.0 | **1.55 (1.41-1.69)** | **<.001** |
| Male | 6883 | 1848 | 50.5 | 60.6 | 79.0 | 21.0 | Reference | - |
| **Ethnicity** |  |  |  |  |  |  |  |  |
| Chinese | 891 | 114 | 11.2 | 6.3 | 88.8 | 11.2 | **2.02 (1.54-2.65)** | **<.001** |
| South Asian | 604 | 69 | 8.3 | 4.6 | 89.0 | 11.0 | **1.80 (1.27-2.56)** | **.001** |
| Other visible minority | 1267 | 155 | 6.7 | 3.8 | 88.8 | 11.2 | **1.89 (1.49-2.40)** | **<.001** |
| Not a visible minority | 30894 | 6439 | 62.1 | 68.7 | 80.3 | 19.7 | Reference | - |
| Other ethnicity | 1916 | 430 | 8.0 | 9.6 | 78.9 | 21.1 | 0.94 (0.80-1.11) | .48 |
| Prefer not to answer | 1144 | 378 | 3.7 | 6.9 | 70.9 | 29.1 | **0.73 (0.59-0.89)** | **.002** |
| **Number of trips outside home** |  |  |  |  |  |  |  |  |
| Once | 20678 | 4109 | 53.2 | 43.5 | 84.7 | 15.3 | **3.10 (2.61-3.68)** | **<.001** |
| 2 times | 10121 | 1610 | 28.2 | 21.2 | 85.7 | 14.3 | **3.04 (2.57-3.61)** | **<.001** |
| 3 times | 3527 | 769 | 10.2 | 12.3 | 78.9 | 21.1 | **1.90 (1.57-2.30)** | **<.001** |
| 4+ times | 2107 | 879 | 7.3 | 18.2 | 64.5 | 35.5 | Reference | - |
| Prefer not to answer/Missing | 283 | 218 | 1.1 | 4.8 | 50.5 | 49.5 | 1.16 (0.73-1.84) | .52 |
| **Destination^b^** |  |  |  |  |  |  |  |  |
| A restaurant, bar, or café | 631 | 102 | 1.9 | 1.8 | 82.8 | 17.2 | **4.65 (2.89-7.48)** | **<.001** |
| A workplace | 4231 | 365 | 14.2 | 6.4 | 90.9 | 9.1 | **8.57 (6.91-10.63)** | **<.001** |
| Another person's home | 838 | 421 | 2.2 | 4.5 | 69.2 | 30.8 | **1.92 (1.49-2.48)** | **<.001** |
| Multiple destinations | 15804 | 2264 | 43.5 | 38.3 | 83.7 | 16.3 | **7.63 (6.51-8.94)** | **<.001** |
| Other destinations /Missing/Prefer not to answer | 4048 | 1358 | 10.5 | 18.1 | 72.4 | 27.6 | **3.10 (2.60-3.68)** | **<.001** |
| Retail including grocery store, pharmacy, liquor store | 8116 | 447 | 20.3 | 5.6 | 94.2 | 5.8 | **14.23 (11.69-17.31)** | **<.001** |
| Park or other public space | 3048 | 2628 | 7.4 | 25.3 | 56.8 | 43.2 | Reference | - |
| **Mode of travel^b^** |  |  |  |  |  |  |  |  |
| I only walked (I did not use transportation) | 4791 | 2445 | 13.6 | 24.9 | 71.2 | 28.8 | Reference | - |
| Other/Missing/Prefer not to answer | 1521 | 367 | 6.2 | 7.9 | 78.1 | 21.9 | **2.56 (1.98-3.31)** | **<.001** |
| Alone in a car | 15544 | 1857 | 40.0 | 24.7 | 88.0 | 12.0 | **2.15 (1.86-2.50)** | **<.001** |
| Bicycle, moped, or motorcycle | 508 | 149 | 1.9 | 2.4 | 77.8 | 22.2 | **1.76 (1.20-2.60)** | **.004** |
| In a car with someone else (not a taxi) | 8753 | 1856 | 22.3 | 24.6 | 80.3 | 19.7 | **1.59 (1.36-1.86)** | **<.001** |
| Multiple modes of travel | 5599 | 911 | 16.0 | 15.5 | 82.3 | 17.7 | **1.97 (1.65-2.35)** | **<.001** |
| **Distance traveled** |  |  |  |  |  |  |  |  |
| Less than 1 km | 4944 | 1443 | 12.8 | 15.2 | 79.2 | 20.8 | Reference | - |
| 1 to 10 km | 22670 | 4059 | 59.5 | 46.4 | 85.2 | 14.8 | 1.07 (0.92-1.23) | .37 |
| 10 to 100 km | 8126 | 1598 | 24.2 | 28.1 | 79.5 | 20.5 | **0.72 (0.60-0.86)** | **<.001** |
| More than 100 km | 652 | 245 | 2.0 | 4.9 | 64.7 | 35.3 | **0.55 (0.40-0.74)** | **<.001** |
| Prefer not to answer/Missing | 324 | 240 | 1.5 | 5.4 | 56.1 | 43.9 | **0.57 (0.37-0.88)** | **.01** |
| **Health Authority** |  |  |  |  |  |  |  |  |
| Fraser | 7339 | 1333 | 26.8 | 24.1 | 83.4 | 16.6 | **1.99 (1.55-2.55)** | **<.001** |
| Interior | 4896 | 1354 | 11.0 | 16.2 | 75.5 | 24.5 | 1.18 (0.92-1.51) | .19 |
| Vancouver Coastal | 1378 | 356 | 3.7 | 5.6 | 75.1 | 24.9 | **2.87 (2.23-3.69)** | **<.001** |
| Vancouver Island | 7079 | 1103 | 20.3 | 14.3 | 86.5 | 13.5 | **2.04 (1.60-2.61)** | **<.001** |
| Northern | 7274 | 1574 | 12.6 | 12.8 | 81.6 | 18.4 | Reference | - |
| Missing | 8750 | 1865 | 25.4 | 27.0 | 81.0 | 19.0 | **1.84 (1.45-2.35)** | **<.001** |
| **Time period** |  |  |  |  |  |  |  |  |
| Sep 4-30, 2020 | 1525 | 422 | 3.6 | 4.6 | 77.9 | 22.1 | Reference | - |
| Oct 1-31, 2020 | 2982 | 745 | 8.8 | 11.1 | 78.0 | 22.0 | 1.16 (0.90-1.49) | .25 |
| Nov 1-30, 2020 | 5140 | 919 | 11.2 | 9.5 | 84.2 | 15.8 | **1.75 (1.39-2.21)** | **<.001** |
| Dec 1-31, 2020 | 3986 | 501 | 10.3 | 5.3 | 89.8 | 10.2 | **3.54 (2.72-4.61)** | **<.001** |
| Jan 1-31, 2021 | 3909 | 630 | 9.7 | 6.0 | 87.9 | 12.1 | **2.97 (2.31-3.82)** | **<.001** |
| Feb 1-28, 2021 | 2278 | 287 | 6.3 | 3.2 | 89.9 | 10.1 | **3.79 (2.85-5.04)** | **<.001** |
| Mar 1-31, 2021 | 1109 | 162 | 3.1 | 2.3 | 85.9 | 14.1 | **2.69 (1.87-3.86)** | **<.001** |
| Apr 1-30, 2021 | 2279 | 267 | 6.3 | 3.3 | 89.7 | 10.3 | **3.90 (2.86-5.31)** | **<.001** |
| May 1-31, 2021 | 1671 | 194 | 5.3 | 2.7 | 90.0 | 10.0 | **3.65 (2.64-5.05)** | **<.001** |
| Jun 1-30, 2021 | 689 | 79 | 2.6 | 1.1 | 91.6 | 8.4 | **4.11 (2.72-6.20)** | **<.001** |
| Jul 1-31, 2021 | 1139 | 271 | 3.4 | 3.7 | 80.2 | 19.8 | 1.17 (0.86-1.59) | .32 |
| Aug 1-31, 2021 | 1331 | 379 | 4.5 | 5.9 | 77.4 | 22.6 | 1.15 (0.85-1.56) | .36 |
| Sep 1-30, 2021 | 1229 | 172 | 4.3 | 2.7 | 87.9 | 12.1 | **3.07 (2.13-4.42)** | **<.001** |
| Oct 1-31, 2021 | 868 | 100 | 2.7 | 1.5 | 89.0 | 11.0 | **2.87 (1.89-4.35)** | **<.001** |
| Nov 1-30, 2021 | 630 | 101 | 1.7 | 1.5 | 83.4 | 16.6 | **1.95 (1.32-2.89)** | **<.001** |
| Dec 1-31, 2021 | 964 | 133 | 2.8 | 2.2 | 84.9 | 15.1 | **2.06 (1.46-2.91)** | **<.001** |
| Jan 1-31, 2022 | 1308 | 200 | 3.8 | 3.0 | 85.3 | 14.7 | **2.21 (1.57-3.11)** | **<.001** |
| Feb 1-28, 2022 | 828 | 193 | 2.6 | 2.8 | 80.7 | 19.3 | **1.46 (1.07-1.99)** | **.02** |
| Mar 1-31, 2022 | 629 | 304 | 1.9 | 4.3 | 67.3 | 32.7 | 0.71 (0.51-1.00) | .049 |
| Apr 1-30, 2022 | 892 | 418 | 1.9 | 5.7 | 60.1 | 39.9 | **0.45 (0.34-0.62)** | **<.001** |
| May 1-31, 2022 | 605 | 413 | 1.4 | 6.0 | 52.2 | 47.8 | **0.32 (0.23-0.43)** | **<.001** |
| Jun 1-30, 2022 | 276 | 299 | 0.7 | 5.2 | 38.1 | 61.9 | **0.18 (0.12-0.26)** | **<.001** |
| Jul 1-31, 2022 | 449 | 396 | 1.2 | 6.5 | 44.8 | 55.2 | **0.31 (0.22-0.44)** | **<.001** |
| **^a^**Face mask usage, Yes N= 36,716, No N= 7,585; ^b^Composite variable (groups merged). Bolded values are statistically significant. | | | | | | | | |

**Table S3.** Characteristics of British Columbia COVID-19 Population Mixing Patterns (BC-Mix) survey respondents by face mask usage in the presence and absence of the provincial mask mandate, September 2020-July 2022

|  | **Mask mandate in effect (N= 30,826)** | | | | | | **Mask mandate not in effect (N= 13,475)** | | | | | |
| --- | --- | --- | --- | --- | --- | --- | --- | --- | --- | --- | --- | --- |
|  | **Face Mask Use: Yes^a^** | **Face Mask Use: No^a^** | **Face Mask Use: Yes^a^** | **Face Mask Use: No^a^** | **Face Mask Use: Yes** | **Face Mask Use: No** | **Face Mask Use: Yes^b^** | **Face Mask Use: No^b^** | **Face Mask Use: Yes^b^** | **Face Mask Use: No^b^** | **Face Mask Use: Yes** | **Face Mask Use: No** |
|  | **n (unweighted)** | **n (unweighted)** | **column % (weighted)** | **column % (weighted)** | **row % (weighted)** | **row % (weighted)** | **n (unweighted)** | **n (unweighted)** | **column % (weighted)** | **column % (weighted)** | **row % (weighted)** | **row % (weighted)** |
| **Age** |  |  |  |  |  |  |  |  |  |  |  |  |
| 18-24 years | 722 | 87 | 7.7 | 7.9 | 87.1 | 12.9 | 373 | 152 | 10.5 | 11.1 | 68.7 | 31.3 |
| 25-34 years | 2281 | 310 | 16.7 | 17.7 | 86.8 | 13.2 | 1178 | 386 | 23.6 | 21.2 | 72.2 | 27.8 |
| 35-44 years | 3708 | 520 | 15.8 | 16.3 | 87.1 | 12.9 | 1449 | 546 | 16.2 | 16.1 | 70.0 | 30.0 |
| 45-54 years | 4647 | 556 | 19.4 | 16.1 | 89.3 | 10.7 | 1762 | 603 | 17.0 | 20.0 | 66.5 | 33.5 |
| 55-64 years | 7003 | 1111 | 18.2 | 19.2 | 86.9 | 13.1 | 2146 | 858 | 13.8 | 13.3 | 70.8 | 29.2 |
| 65-74 years | 6571 | 1047 | 16.8 | 17.4 | 87.1 | 12.9 | 2246 | 839 | 14.4 | 13.8 | 70.8 | 29.2 |
| 75+ years | 1956 | 307 | 5.4 | 5.2 | 87.9 | 12.1 | 674 | 263 | 4.6 | 4.5 | 70.7 | 29.3 |
| **Sex** |  |  |  |  |  |  |  |  |  |  |  |  |
| Female | 21665 | 3061 | 48.5 | 43.2 | 88.7 | 11.3 | 8168 | 2676 | 52.1 | 36.0 | 77.1 | 22.9 |
| Male | 5223 | 877 | 51.5 | 56.8 | 86.3 | 13.7 | 1660 | 971 | 47.9 | 64.0 | 63.6 | 36.4 |
| **Ethnicity** |  |  |  |  |  |  |  |  |  |  |  |  |
| Chinese | 594 | 60 | 10.2 | 6.2 | 92.0 | 8.0 | 297 | 54 | 13.6 | 6.4 | 83.1 | 16.9 |
| South Asian | 433 | 28 | 8.6 | 3.9 | 94.0 | 6.0 | 171 | 41 | 7.3 | 5.3 | 76.3 | 23.7 |
| Other visible minority | 869 | 68 | 6.1 | 3.3 | 92.9 | 7.1 | 398 | 87 | 8.2 | 4.3 | 81.8 | 18.2 |
| Not a visible minority | 22672 | 3388 | 62.8 | 70.8 | 86.1 | 13.9 | 8222 | 3051 | 60.2 | 66.8 | 67.7 | 32.3 |
| Other ethnicity | 1426 | 214 | 8.2 | 9.4 | 85.8 | 14.2 | 490 | 216 | 7.5 | 9.8 | 64.2 | 35.8 |
| Prefer not to answer | 894 | 180 | 4.0 | 6.5 | 81.3 | 18.7 | 250 | 198 | 3.0 | 7.3 | 49.0 | 51.0 |
| **Education** |  |  |  |  |  |  |  |  |  |  |  |  |
| Below High School | 499 | 58 | 2.5 | 1.9 | 90.2 | 9.8 | 93 | 48 | 1.1 | 1.7 | 59.8 | 40.2 |
| Below Bachelor's degree | 10284 | 1567 | 34.8 | 35.0 | 87.4 | 12.6 | 3550 | 1379 | 31.8 | 35.2 | 67.8 | 32.2 |
| University degree | 9455 | 1433 | 35.3 | 33.2 | 88.1 | 11.9 | 4142 | 1243 | 44.3 | 33.3 | 75.6 | 24.4 |
| Prefer not to answer/Missing | 6650 | 880 | 27.3 | 29.9 | 86.4 | 13.6 | 2043 | 977 | 22.8 | 29.8 | 64.1 | 35.9 |
| **Employment status** |  |  |  |  |  |  |  |  |  |  |  |  |
| Employed full-time | 7383 | 852 | 32.2 | 27.4 | 89.2 | 10.8 | 3019 | 906 | 37.5 | 31.9 | 73.2 | 26.8 |
| Employed part-time | 1980 | 201 | 7.0 | 5.1 | 90.5 | 9.5 | 827 | 226 | 7.8 | 5.1 | 78.1 | 21.9 |
| Self-employed | 1700 | 298 | 7.0 | 7.1 | 87.3 | 12.7 | 568 | 264 | 5.4 | 8.1 | 61.0 | 39.0 |
| Unemployed | 723 | 124 | 3.8 | 4.0 | 86.8 | 13.2 | 269 | 107 | 3.8 | 4.4 | 66.6 | 33.4 |
| Full-time parent, homemaker | 454 | 102 | 1.3 | 2.1 | 81.4 | 18.6 | 194 | 97 | 1.6 | 2.0 | 64.7 | 35.3 |
| Retired | 7341 | 1366 | 18.0 | 20.2 | 86.2 | 13.8 | 2552 | 973 | 15.6 | 14.3 | 71.8 | 28.2 |
| Student/Pupil | 286 | 43 | 2.2 | 2.3 | 87.3 | 12.7 | 189 | 51 | 3.8 | 2.8 | 75.8 | 24.2 |
| Long-term sick/disabled | 479 | 68 | 1.7 | 1.7 | 87.1 | 12.9 | 191 | 58 | 1.8 | 1.5 | 73.5 | 26.5 |
| Prefer not to answer/Missing | 6542 | 884 | 26.8 | 30.2 | 86.1 | 13.9 | 2019 | 965 | 22.6 | 29.7 | 63.9 | 36.1 |
| **Occupation** |  |  |  |  |  |  |  |  |  |  |  |  |
| Essential workers | 5787 | 755 | 24.2 | 23.2 | 87.9 | 12.1 | 2203 | 781 | 24.2 | 27.1 | 67.5 | 32.5 |
| Non-essential workers | 7203 | 985 | 25.7 | 23.2 | 88.6 | 11.4 | 3043 | 913 | 31.6 | 22.5 | 76.6 | 23.4 |
| Others | 1781 | 245 | 7.2 | 5.7 | 89.9 | 10.1 | 677 | 255 | 7.1 | 7.7 | 68.2 | 31.8 |
| Do not work | 5433 | 1051 | 15.4 | 17.1 | 86.3 | 13.7 | 1849 | 698 | 13.8 | 11.5 | 73.6 | 26.4 |
| Prefer not to answer/Missing | 6684 | 902 | 27.6 | 30.8 | 86.2 | 13.8 | 2056 | 1000 | 23.4 | 31.1 | 63.6 | 36.4 |
| **Health Authority** |  |  |  |  |  |  |  |  |  |  |  |  |
| Fraser Health | 5378 | 647 | 26.7 | 21.6 | 89.6 | 10.4 | 1961 | 686 | 27.1 | 26.3 | 70.6 | 29.4 |
| Interior Health | 3724 | 715 | 11.8 | 16.5 | 83.3 | 16.7 | 1172 | 639 | 9.1 | 15.9 | 57.2 | 42.8 |
| Northern Health | 1116 | 186 | 4.3 | 5.8 | 83.7 | 16.3 | 262 | 170 | 2.3 | 5.4 | 49.6 | 50.4 |
| Vancouver Coastal Health | 4784 | 593 | 18.4 | 15.5 | 89.2 | 10.8 | 2295 | 510 | 25.5 | 13.2 | 81.9 | 18.1 |
| Vancouver Island Health | 5180 | 867 | 12.1 | 13.5 | 86.2 | 13.8 | 2094 | 707 | 13.8 | 12.2 | 72.4 | 27.6 |
| Missing | 6706 | 930 | 26.7 | 27.0 | 87.3 | 12.7 | 2044 | 935 | 22.1 | 26.9 | 65.7 | 34.3 |
| ^a^Face mask usage, Yes N= 26,888, No N= 3,938; ^b^Face mask usage, Yes N= 9,828, No N= 3,647 | | | | | | | | | | | | |

**Table S4.** Odds ratios for face mask usage among British Columbia COVID-19 Population Mixing Patterns (BC-Mix) survey respondents in the presence or absence of the provincial mask mandate, September 2020-July 2022.

|  | **Overall (N= 44,301)^a^** | | **Mask mandate in effect  (N= 30,826)^b^** | | **Mask mandate not in effect  (N= 13,475)^c^** | |
| --- | --- | --- | --- | --- | --- | --- |
|  | **Adjusted Odds Ratio (95% CI)** | ***P*-value** | **Adjusted Odds Ratio (95% CI)** | ***P*-value** | **Adjusted Odds Ratio (95% CI)** | ***P*-value** |
| **Age** |  |  |  |  |  |  |
| 18-24 years | 0.87 (0.66-1.14) | .30 | 0.95 (0.65-1.39) | .79 | 0.83 (0.55-1.25) | .37 |
| 25-34 years | **0.79 (0.64-0.98)** | **.03** | **0.69 (0.53-0.89)** | **.01** | **0.89 (0.63-1.24)** | **.48** |
| 35-44 years | 0.81 (0.65-1.00) | .05 | 0.79 (0.62-1.02) | .07 | 0.82 (0.58-1.15) | .25 |
| 45-54 years | **0.80 (0.66-0.99)** | **.04** | 0.90 (0.71-1.14) | .39 | **0.69 (0.49-0.96)** | **.03** |
| 55-64 years | **0.74 (0.61-0.90)** | **.003** | **0.71 (0.57-0.88)** | **.002** | 0.79 (0.58-1.08) | .15 |
| 65-74 years | 0.87 (0.72-1.06) | .16 | 0.82 (0.66-1.01) | .06 | 0.93 (0.68-1.26) | .64 |
| 75+ years | Reference | - | Reference | - | Reference | - |
| **Sex** |  |  |  |  |  |  |
| Female | **1.53 (1.40-1.67)** | **<.001** | **1.27 (1.12-1.43)** | **<.001** | **1.90 (1.66-2.16)** | **<.001** |
| Male | Reference | - | Reference | - | Reference | - |
| **Ethnicity** |  |  |  |  |  |  |
| Chinese | **1.89 (1.45-2.46)** | **<.001** | **1.51 (1.09-2.10)** | **.01** | **2.13 (1.47-3.09)** | **<.001** |
| South Asian | **1.90 (1.34-2.68)** | **<.001** | **2.21 (1.37-3.57)** | **.001** | 1.59 (0.98-2.58) | .06 |
| Other visible minority | **1.98 (1.55-2.51)** | **<.001** | **1.90 (1.35-2.68)** | **<.001** | **2.00 (1.44-2.76)** | **<.001** |
| Not a visible minority | Reference | - | Reference | - | Reference | - |
| Other ethnicity | 0.92 (0.78-1.08) | .32 | 0.98 (0.78-1.22) | .84 | 0.85 (0.67-1.07) | .17 |
| Prefer not to answer | **0.71 (0.58-0.87)** | **<.001** | 0.85 (0.64-1.12) | .24 | **0.59 (0.43-0.81)** | **.001** |
| **Number of trips outside home** |  |  |  |  |  |  |
| Once | **3.27 (2.76-3.88)** | **<.001** | **4.19 (3.31-5.30)** | **<.001** | **2.58 (2.00-3.31)** | **<.001** |
| 2 times | **3.16 (2.67-3.74)** | **<.001** | **3.35 (2.65-4.24)** | **<.001** | **3.02 (2.37-3.84)** | **<.001** |
| 3 times | **1.88 (1.55-2.27)** | **<.001** | **2.94 (2.23-3.86)** | **<.001** | **1.35 (1.03-1.77)** | **.03** |
| 4+ times | Reference | - | Reference | - | Reference | - |
| Prefer not to answer/Missing | 1.12 (0.73-1.72) | .60 | 1.04 (0.62-1.76) | .87 | 1.11 (0.56-2.18) | .77 |
| **Destination^d^** |  |  |  |  |  |  |
| A restaurant, bar, or café | **4.43 (2.85-6.87)** | **<.001** | **7.35 (4.23-12.78)** | **<.001** | **2.81 (1.50-5.26)** | **.001** |
| A workplace | **7.89 (6.37-9.77)** | **<.001** | **9.38 (7.03-12.52)** | **<.001** | **5.68 (4.04-7.98)** | **<0001** |
| Another person's home | **1.87 (1.45-2.41)** | **<.001** | **1.95 (1.43-2.68)** | **<.001** | **1.49 (0.98-2.26)** | **.06** |
| Multiple destinations | **6.94 (5.95-8.09)** | **<.001** | **9.06 (7.47-10.99)** | **<.001** | **4.55 (3.52-5.89)** | **<.001** |
| Other destinations/Missing/Prefer not to answer | **2.89 (2.44-3.43)** | **<.001** | **2.71 (2.24-3.28)** | **<.001** | **2.72 (1.99-3.72)** | **<.001** |
| Retail including grocery store, pharmacy, liquor store | **13.08 (10.75-15.92)** | **<.001** | **19.94 (14.86-26.77)** | **<.001** | **7.71 (5.68-10.46)** | **<.001** |
| Park or other public space | Reference | - | Reference | - | Reference | - |
| **Mode of travel^d^** |  |  |  |  |  |  |
| I only walked (I did not use transportation) | Reference | - | Reference | - | Reference | - |
| Other/Missing/Prefer not to answer | **2.33 (1.78-3.05)** | **<.001** | **2.76 (2.01-3.80)** | **<.001** | **1.95 (1.30-2.91)** | **.001** |
| Alone in a car | **2.13 (1.84-2.47)** | **<.001** | **2.45 (2.03-2.95)** | **<.001** | **1.76 (1.37-2.27)** | **<.001** |
| Bicycle, moped, or motorcycle | **1.76 (1.20-2.60)** | **.004** | **1.85 (1.09-3.14)** | **.02** | **1.53 (0.87-2.69)** | **.138** |
| In a car with someone else (not a taxi) | **1.57 (1.34-1.83)** | **<.001** | **1.52 (1.26-1.84)** | **<.001** | **1.53 (1.18-1.98)** | **.001** |
| Multiple modes of travel | **1.95 (1.64-2.33)** | **<.001** | **1.84 (1.46-2.33)** | **<.001** | **1.97 (1.50-2.58)** | **<.001** |
| **Distance traveled** |  |  |  |  |  |  |
| Less than 1 km | Reference | - | Reference | - | Reference | - |
| 1 to 10 km | 1.08 (0.94-1.24) | .30 | **1.19 (1.01-1.41)** | **.04** | 0.93 (0.72-1.19) | .55 |
| 10 to 100 km | **0.72 (0.61-0.86)** | **<.001** | **0.71 (0.57-0.89)** | **.003** | **0.68 (0.51-0.91)** | **.01** |
| More than 100 km | **0.55 (0.41-0.73)** | **<.001** | **0.44 (0.30-0.65)** | **<.001** | **0.60 (0.39-0.92)** | **.02** |
| Prefer not to answer/Missing | **0.50 (0.33-0.75)** | **<.001** | **0.43 (0.27-0.70)** | **.001** | 0.52 (0.26-1.05) | .07 |
| **Health Authority** |  |  |  |  |  |  |
| Fraser | **1.98 (1.54-2.55)** | **<.001** | **1.76 (1.28-2.42)** | **<.001** | **2.48 (1.65-3.74)** | **<.001** |
| Interior | 1.19 (0.93-1.53) | .17 | 1.07 (0.78-1.47) | .68 | 1.41 (0.94-2.13) | .10 |
| Vancouver Coastal | **2.95 (2.28-3.81)** | **<.001** | **2.03 (1.47-2.79)** | **<.001** | **4.66 (3.08-7.05)** | **<.001** |
| Vancouver Island | **2.02 (1.58-2.59)** | **<.001** | **1.49 (1.09-2.03)** | **.01** | **3.06 (2.04-4.57)** | **<.001** |
| Northern | Reference | - | Reference | - | Reference | - |
| Missing | **1.82 (1.42-2.33)** | **<.001** | **1.70 (1.24-2.32)** | **<.001** | **2.19 (1.46-3.28)** | **<.001** |
| **Mask mandate in effect** |  |  |  |  |  |  |
| No | Reference | - | - | - | - | - |
| Yes | **3.68 (3.33-4.05)** | **<.001** | - | - | - | - |
| ^a^Face mask usage, Yes N= 36,716, No N= 7,585; ^b^Face mask usage, Yes N= 26,888, No N= 3,938; ^c^Face mask usage, Yes N= 9,828, No N= 3,647;.^d^Composite variable (groups merged). Bolded values are statistically significant. | | | | | | |

**Figure S1.** Face mask usage patterns among BC-Mix survey respondents, September 2020-July 2022. (A) Location of face mask use: Percentages calculated independently for each option provided. (B) Duration of face mask use.

**
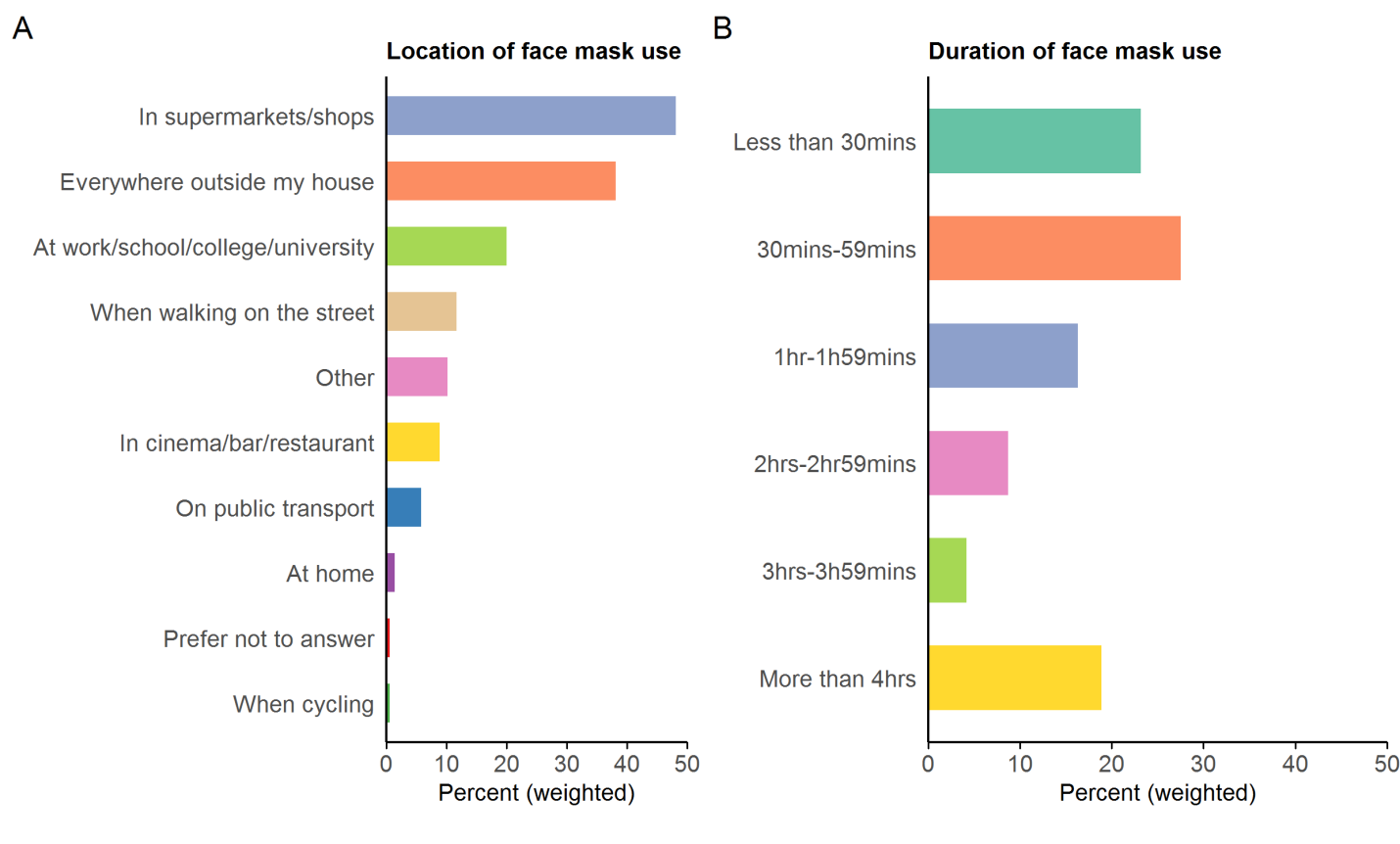
**

**Figure S2.** Travel patterns of BC-Mix survey respondents by face mask use, September 2020-July 2022. (A) Number of trips taken outside the home. (B) Destination: Percentages calculated independently for each option provided. (C) Mode of travel: Percentages calculated independently for each option provided.


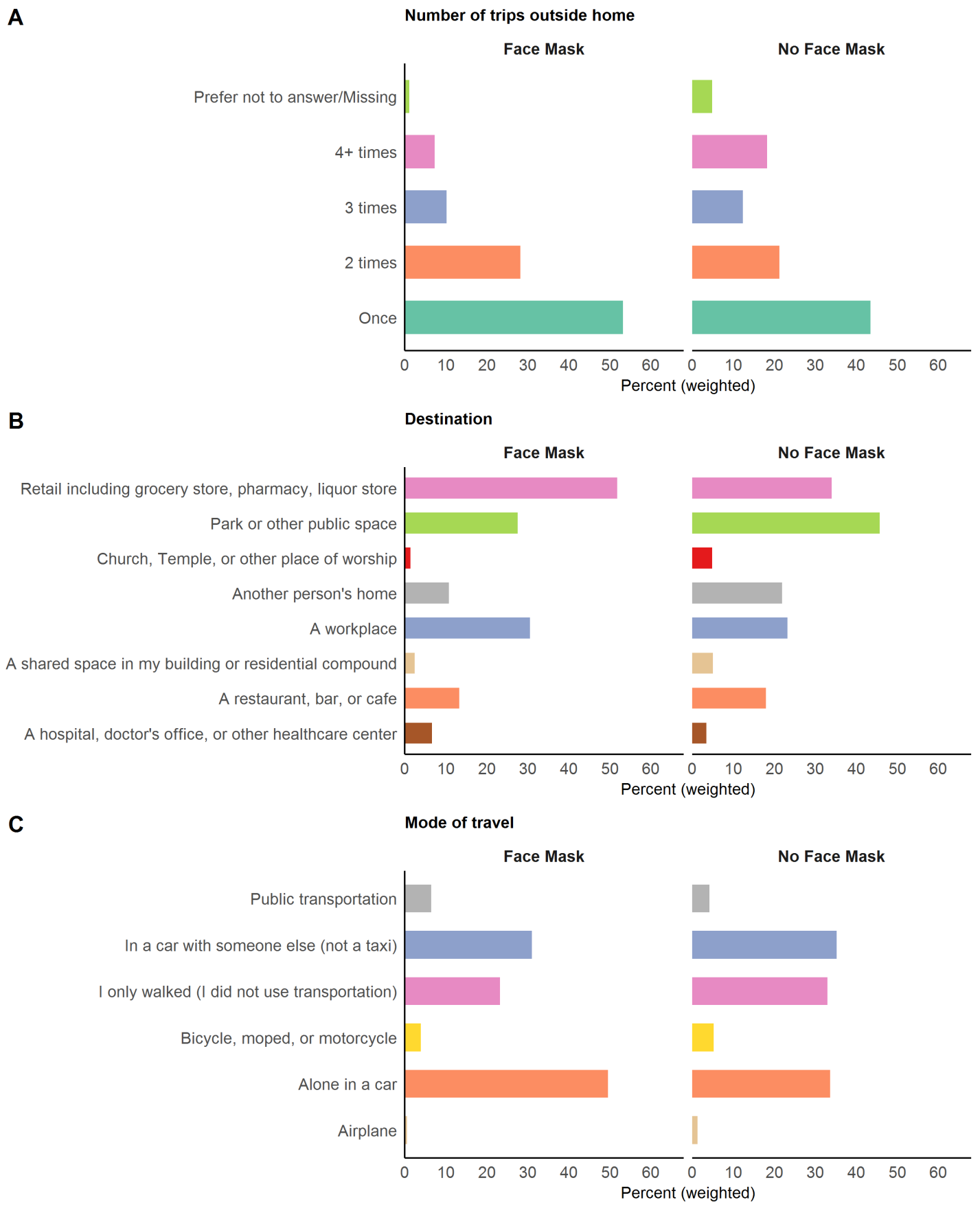

Supplement: Multimedia Appendix 1 [file publichealth_v9i1e42616_app1.docx]
